# Supplementary material for: PRC2 activates interferon-stimulated genes indirectly by repressing miRNAs in glioblastoma
Source: PLoS One. 2019 Sep 12;14(9):e0222435. doi: 10.1371/journal.pone.0222435 (PMC6742368; doi:10.1371/journal.pone.0222435)
Supplement: S1 Table — (PDF) [file pone.0222435.s001.pdf]

Supplementary Table 1. Primers for the amplification of 3' UTRs of EZH2-activated genes

**Gene**	**Primers for amplification of 3' UTRs**
IL15RA	TAAGCACTCGAGAACTCGGGGAAACCAGCCCA
	TAAGCAGCGGCCGCCTGAATCCTTCAATGGAGAGGATTCGCTG
HMGA2	TAAGCACTCGAGCACAGGGGACACAGCTTAACAATGC
	TAAGCAGCGGCCGCAGATTTCGCTCCTCCCACCTCA
MX1	TAAGCACTCGAGCCACACTCTGTCCAGCCCC
	TAAGCAGCGGCCGCAGAGGAGACAGGGCTCCGAC
IFIT2	TAAGCACTCGAGTTCTGAGGCTTTGCATGTCTTGGC
	TAAGCAGCGGCCGCGAACTTAGCACATTACTGGCTATGCAGG
OAS3	TAAGCACTCGAGAACATGCTGAGATTCTGCATCCCCACA
	TAAGCAGCGGCCGCCTGCTAAGAAAGGGAGGTGGGATCCG
FMNL1	TAAGCACTCGAGATCTGCGGAACCAGCCCTA
	TAAGCAGCGGCCGCAGCCGACGCGGATCTTTTGC
AXL	TAAGCACTCGAGGACAACCCTCCACCTGGTACT
	TAAGCAGCGGCCGCGAGGAGACAGAAGCTCGTTGAT
PTPRF	TAAGCACTCGAGCTACCGCTCCCCTCTCCT
	TAAGCAGCGGCCGCGACAGAGTCCTGGTTTGTAAGAAATCTG
